# ID 242 - Prioridades de Publicação de Epidemias, Incluindo Respectivas Tecnologias em Saúde, Propostas para Eliminação pelos Objetivos de Desenvolvimento Sustentável em Periódicos Farmacêuticos

**DOI:** 10.5327/2237-9622.2025.v34s1.242

**Published:** 2025-11-25

**Authors:** Julia Soto Rizzato, Marcus Tolentino Silva, Tais Freire Galvão

**Keywords:** desenvolvimento sustentável, publicação periódica, ciência da informação, pesquisa científica e desenvolvimento tecnológico, prioridade de pesquisa

## Abstract

**Introdução:**

A publicação de pesquisas em periódicos é a principal forma de comunicação científica. Os temas publicados nos periódicos são reflexo do interesse em pesquisa na área e do financiamento disponível. As epidemias da meta 3.3 dos Objetivos de Desenvolvimento Sustentável (ODS) – aids, malária e tuberculose – ainda hoje causam grande impacto no mundo, necessitando de novas tecnologias em saúde para melhor enfrentamento e atingimento da meta. Monitorar a publicação sobre o ODS 3.3, o que inclui as tecnologias em saúde envolvidas no seu enfrentamento, permitiria verificar como tais temas e a prioridade de pesquisa e publicação se encontram para traçar estratégias de promoção à pesquisa e ao fomento pertinentes. A presente pesquisa buscou avaliar as prioridades de publicações sobre epidemias a serem erradicadas até 2030, segundo a meta 3.3 dos ODS (aids, tuberculose e malária) em periódicos farmacêuticos.

**Método:**

Este estudo transversal incluiu os periódicos listados na (JCR) em 2022. O desfecho primário foi a prioridade de publicação sobre aids, malária e tuberculose, definida como ≥3% de publicações sobre o tema. Após a exportação de lista dos periódicos com indicadores bibliométricos disponível no JCR, o total de publicações em cada periódico até 2022 e o número de publicações sobre o ODS 3.3 foram identificados por meio do, via EndNote 20. Adotou-se ponto de corte de ≥15% de artigos na modalidade acesso aberto dourado como acesso aberto. O país dos editores-chefe foi coletado no website de cada periódico e classificados como baixa e média ou alta renda, de acordo com o Banco Mundial. Regressão de Poisson foi empregada para calcular razão de prevalências (RP) e intervalo de confiança de 95% (IC 95%) do desfecho pelas características dos periódicos.

**Resultados:**

De 362 periódicos elegíveis, 354 foram incluídos no estudo (83 do grupo [ESCI] e 271 do ), 8 não continham informação sobre a origem dos editores (n=4) ou foram descontinuados (n=4). A maioria dos periódicos iniciou-se entre 1991 e 2010 (39,4%) e era de acesso aberto (50,7%). Periódicos iniciados após 2011 tiveram mais editores de países em desenvolvimento (34,5%; p<0,001) e maior prioridade de publicação sobre o ODS 3.3 (RP=2,32; IC 95%: 1,47-3,66). Editores de países de baixa e média renda foram minoria (n=61; 17,3%), com maioria de seus periódicos pertencentes ao grupo ESCI (39,0%; p<0,001) e com acesso aberto (22,9%; p=0,005); o fator de impacto foi menor nesses periódicos (2,33±2,63 versus 4,10±7,39; p=0,066). O desfecho foi maior em periódicos chefiados por editores de países emergentes (RP=1,57; IC 95%: 1,10-2,23). Não foram encontradas associações entre continentes, categoria do JCR, quartil de fator de impacto e número de artigos publicados por ano (p>0,05).

**Conclusão:**

Periódicos fundados mais recentemente e com editores de países em desenvolvimento publicaram mais artigos sobre o ODS 3.3. Aumentar a diversidade do corpo editorial dos periódicos científicos pode ser uma importante estratégia no caminho para a divulgação de tecnologias em saúde voltadas ao enfrentamento dessas doenças negligenciadas e para o alcance dos ODS até 2030.

